# The landscape mapping of single-cell sequencing in the research progression and hotspots of liver cancer based on systematical analysis

**DOI:** 10.1097/MD.0000000000050022

**Published:** 2026-08-21

**Authors:** Wenbiao Chen, Feng Xiong, Haitao Li

**Affiliations:** a Department of Gastroenterology, Shenzhen People’s Hospital, The Second Clinical Medical College, Jinan University, The First Affiliated Hospital, Southern University of Science and Technology, Shenzhen, China; b Clinical Medical Research Center, Shenzhen People’s Hospital, The Second Clinical Medical College, Jinan University, The First Affiliated Hospital, Southern University of Science and Technology, Shenzhen, China.

**Keywords:** bibliometric analysis, cited journals, cited references, liver cancer, single-cell sequencing

## Abstract

**Background:**

Single-cell sequencing technology is a new research method which is greatly applied to the pathogenesis and clinical research of liver cancer. The aim of the study is to map the research levels, research hotspots, and progressive trends in the field of single-cell sequencing on liver cancer.

**Methods:**

The articles related to liver cancer and single-cell sequencing were collected from Web of Science Core Collection database, and then bibliometric methods including CiteSpace, VOSviewer, and the R package Bibliometric were conducted to analyze the knowledge mapping of nation, instruction, author, reference and keyword as well as their relationships.

**Results:**

379 articles about single-cell sequencing on liver cancer were collected between 2017 and March 2024, and the number of articles is still significantly rising. China and its domestic institutions are the hot areas for this research field, and establishing extensive cooperative relationships. The most cited journals are still authoritative journals such as *Cell* and *Hepatology*. The co-citation relationships among journals revealed that different journals focus on various research content. The hot keywords in recent years are hepatocellular carcinoma and single-cell RNA sequencing, whereas each time period has different research keywords. The highly cited and strong citation burst references are “Landscape of Infiltrating T Cells in Liver Cancer Revealed by Single-Cell Sequencing” published by Zhang et al which provided a seminal framework for subsequent research in the field.

**Conclusion:**

The comprehensive overview of the current status and trends of research on single-cell sequencing on liver cancer will prove beneficial for the progression of this field.

## 1. Introduction

Liver cancer is one of the most common malignant tumors in the world, and its morbidity and mortality are still high. Most patients with liver cancer are diagnosed at an advanced stage and have thus lost the opportunity for optimal surgical intervention.^[1]^ Recently, the treatment of targeted drugs and the use of immunosuppressants has brought substantial progress to the diagnosis and treatment of liver cancer. However, the heterogeneity of tumors, immune resistance, and individual differences limit the widespread use of novel therapies.^[2]^ The research of immunosuppressants is inseparable from the understanding of the composition of immune cell and function of tumor immune microenvironment (TIME)^[3]^; the treatment of targeted drugs for liver cancer requires understanding the evolution of tumor cells and altered genetic information^[4]^; and individual differences in tolerance to targeted drugs need a clear understanding of tumor cell heterogeneity.^[5]^ The above description indicates that it is of great significance to study the etiology of liver cancer from the traditional morphology to the cellular, molecular and gene levels. Therefore, understanding the cell map and molecular composition, heterogeneity, and TIME of liver cancer are essential for the biological research of liver cancer and the development of new therapeutic strategies.

Single-cell sequencing technology has gained substantial prominence among researchers and clinicians since its introduction in 2009.^[6]^ Compared to previous oncology studies that heavily relied on sequencing results from large tissue samples composed of numerous cells and only the average gene expression of a group of cells was obtained, single-cell sequencing technology could accurately analyze the gene expression level of each cell and maximize the heterogeneity within the sample to capture rich biological information.^[7]^ The core mechanism of single-cell sequencing lies in that it breaks through the limitation of traditional “population sequencing” that can only obtain the average signal of cells by isolating and lysing individual cells and amplifying the entire genome of extremely trace amounts of mRNA.^[8]^ Specifically, single-cell sequencing isolates individual cells and amplifies their mRNA to profile gene expression at single-cell resolution. This technique identifies rare cell subsets and infers cellular trajectories via RNA velocity. In liver cancer, it has mapped T cell infiltration landscapes, revealing immune cell functional states within the tumor microenvironment and informing immune evasion mechanisms and immunotherapy development.^[9]^ In 2009, *Tang et al* reported for the first time the sequencing analysis of single-cell transcripts.^[6]^ Since then, the construction methods of single-cell mRNA deep sequencing libraries have been continuously optimized, and the throughput has been continuously improved.^[10,11]^ Thus, single-cell sequencing has gradually become an effective method to study the differences in transcription groups between liver cancer cells and reveal cell types, subtypes, states and development tracks, which is helpful to explore the pathogenesis of liver cancer and conduct accurate liver cancer subtypes to guide clinical treatment.^[12]^

At present, several reviews have summarized the application of single-cell sequencing and its significant role in the field of liver,^[12]^ but lack discussion on research hotspots and trends. Bibliometrics is a branch of information science that uses quantitative analysis and statistics to study patterns in the academic literature to measure and evaluate the performance and impact of individuals, institutions, countries, and even entire disciplines.^[13]^ It can also be used to identify emerging research areas and monitor research trends.^[13]^ Here, we performed a systematic bibliometric analysis to investigate the articles related to single-cell sequencing and liver cancer, mapping the influence of individuals, institutions and states in this field, and predicting the research hotspots, trends and evolution using keywords, citations, and references.

## 2. Material and methods

### 2.1. Data source and search strategy

All literature data in this study were sourced from the Web of Science Core Collection database, which encompasses academic publications from over 200 different disciplines globally. Numerous researchers in the field of bibliometrics have validated its effectiveness. The timeframe for literature analysis in this article spans from January 1, 2017, to March 15, 2024. The retrieval strategy used is as follows: TS=(“liver cancer” OR “Liver Neoplasm” OR “Liver tumor” OR “liver tumors” OR “Hepatocellular Cancers” OR “intrahepatic cholangiocarcinoma” OR “hepatocellular cancers” OR “hepatoma” OR “Liver Carcinoma” OR “Hepatic tumor” OR “Hepatic cancer” OR “Cancer of Liver” OR “cancer of the liver” OR “hepatic neoplasms” OR “Neoplasm of liver” OR “liver and intrahepatic bile duct carcinoma” OR “liver and intrahepatic biliary tract cancer” OR “Tumor of liver” OR “liver malignant tumors” OR “Neoplasm of the liver” OR “Hepatocellular Carcinoma (HCC)” OR “liver cell carcinoma”) AND TS=(“single cell sequencing” OR “Single-cell transcriptome” OR “Single-cell RNA-seq” OR “single-cell transcriptomic” OR “single-cell transcriptomics” OR “Single-Cell RNA Sequencing” OR “single-cell multiomics sequencing”). The retrieval and research processes were illustrated in Figure S1, Supplemental Digital Content 1. Only literature in the English language was included in the analysis, and after excluding documents classified as letters, comments, and meetings, a total of 379 relevant articles were obtained. This study was approved by the Ethics Committee of Shenzhen People’s Hospital.

### 2.2. Data analysis

After verifying the correctness of the exported data, the filtered and optimized original dataset will be exported in TXT file format, including important information such as titles, authors, keywords, affiliations, countries/regions, citations, journals, and publication dates. Subsequently, Microsoft Office Excel 2021, VOSviewer (version 1.6.18), CiteSpace (version 6.1.R6), and the R package “Bibliometrix” will be used as the main tools for data analysis and visualization.

VOSviewer is a bibliometric analysis software used for data extraction and processing. In this study, VOSviewer is utilized for analyzing the distribution of countries/regions, institutions, author collaboration, and distribution, as well as the distribution and collaboration of keywords.^[14,15]^ Additionally, CiteSpace is employed for analyzing the co-occurrence of journals, cooperation, and distribution of references, document bursts through double mapping overlay.^[16,17]^ Furthermore, an open-source R package called “Bibliometrix,” developed by Massimo Aria and Corrado Cuccurullo, is used for visualizing the important evolutionary trends of keywords.^[18]^

## 3. Results

### 3.1. Publication and citation analysis

**Figure 1.**
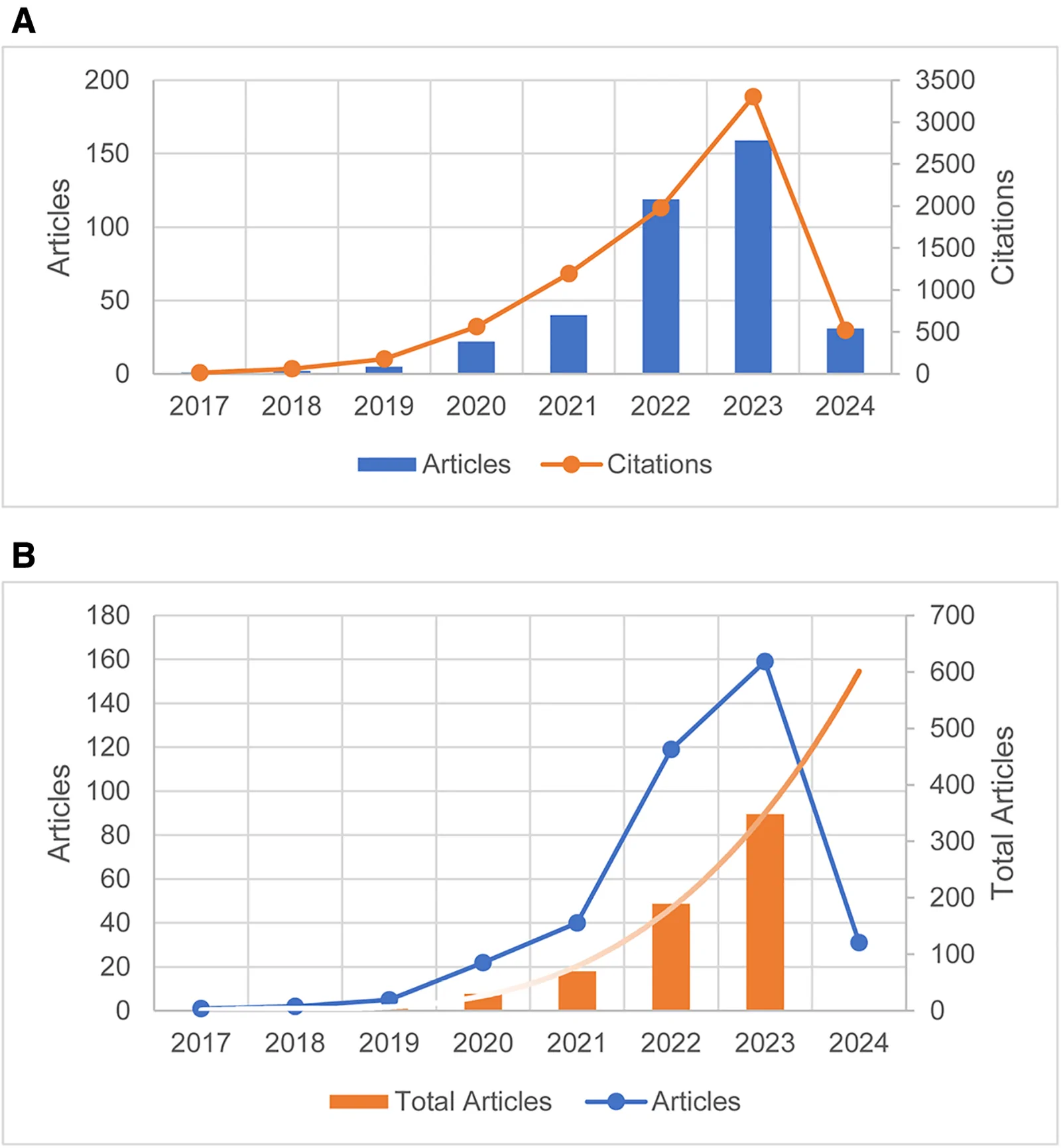
Trends in the published articles and citation counts of liver cancer and single-cell sequencing researches. (A) The annual publication quantity and citation frequency of research on liver cancer and single-cell sequencing from 2017 to 2024. (B) The annual publication quantity, cumulative publication quantity, and their polynomial fitting curves for liver cancer and single-cell sequencing from 2017 to 2024.

A total of 357 articles and their corresponding citations related to single-cell sequencing on liver cancer were retrieved from 2017 to March 15, 2024 (Fig. 1A). It is evident that both the annual number of publications and citations have shown a clear upward trend overall, and this trend is still ongoing, particularly with an increasing magnitude in the number of citations each year. However, as the data collection for 2024 was completed only until mid-March, the related numerical values for 2024 are relatively lower, failing to fully reflect the total volume of publications and citations. Therefore, the peaks in the number of publications and citations so far have occurred in 2023 (159 publications; 348 citations). Additionally, to predict the publication trend of the article in the future, this study further conducted a polynomial fitting of the cumulative number of publications over the years. The fitting equation is *y* = −0.1477 × ^4^ + 5.1136 × ^3^−29.208 × ^2^ + 59.459x–35, with a fitting goodness of R^2^ = 0.9985. This graph allows for the prediction of further growth in the number of publications in this field, indicating the expectation of even more vigorous research activity in this field in 2024 and beyond (Fig. 1B).

### 3.2. Countries/regions and cooperation analysis

If the research results of publications and citation frequency serve as manifestations of the development history and future research vitality of the field, then analyzing the country/region of origin information of relevant publications is an important means to visualize the spatial distribution of research outcomes in the field and the global cooperation relationships. Therefore, the publication of countries/regions and collaborative relationships were investigated. As depicted in Figure S2A, Supplemental Digital Content 2, the relationships among these countries in the form of a chord diagram, where each country is represented by colored bands, and the width of the lines connecting them is positively correlated with the strength of collaboration between countries. It can be observed that China is the country with the most extensive collaboration with other countries, covering approximately half of the global academic exchanges between nations, indicating significant connections with multiple countries. Following China, the United States, Germany, France, and Japan are among the prominent collaborators. A more intuitive geographic distribution is presented in Figure S2B, Supplemental Digital Content 2, where the most significant connections (i.e., cooperation relationships) are observed between China and the United States, with Europe also establishing numerous connections worldwide. This information on collaboration relationships provides us with a clearer academic relationship view on a global scale, and these close academic exchanges will further promote the development of the field.

### 3.3. Authors and cooperation analysis

**Figure 2.**
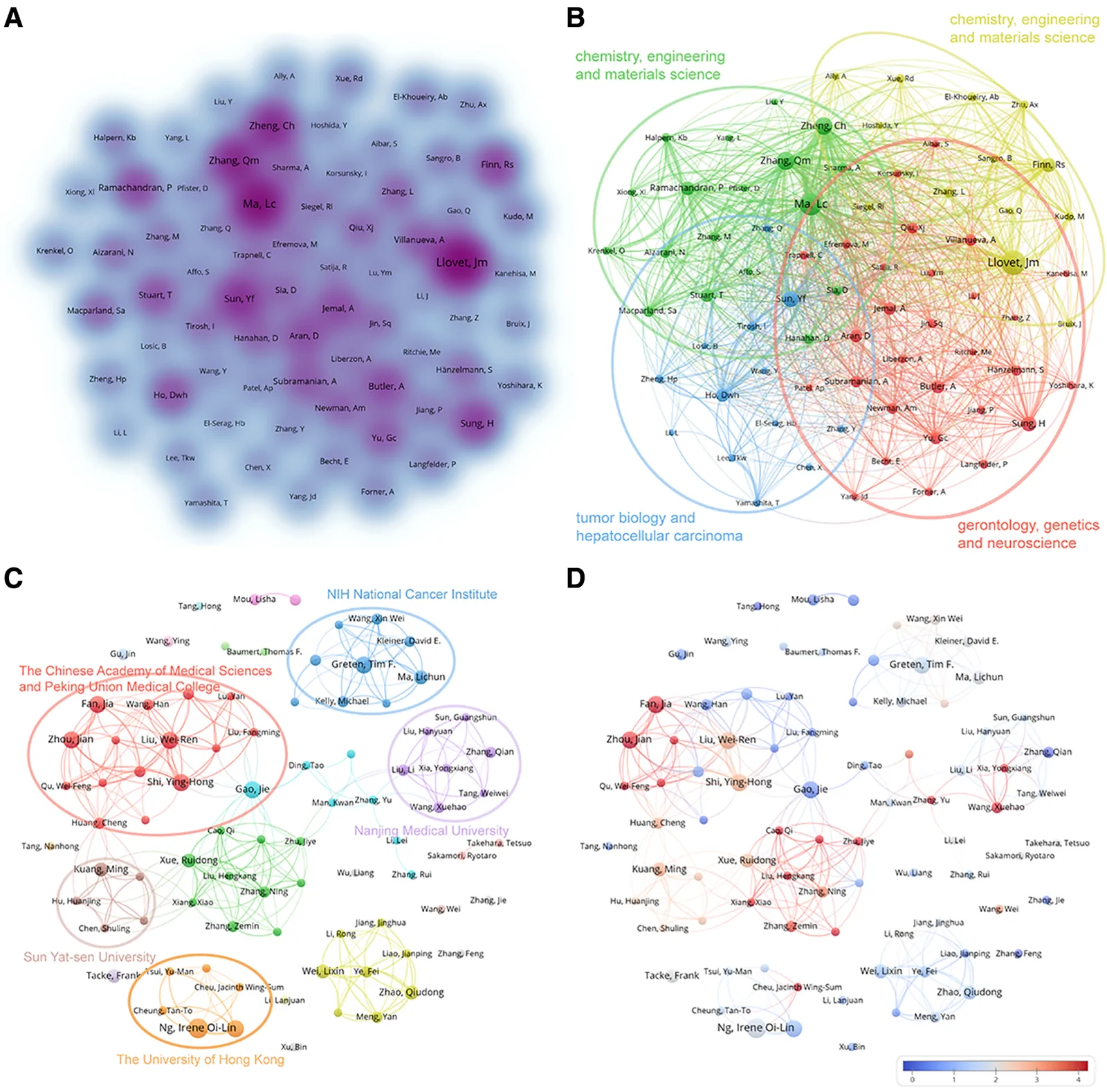
Network visualization of author publication volume, collaboration, and citation relationships in the field of liver cancer and single-cell sequencing. (A) The map overlays authors’ publication volume, where the intensity of color depth indicates the volume of publications. (B) The diagram illustrates co-cited authors in research on liver cancer and single-cell sequencing, with node size indicating citation frequency. This visualization, created using VOSViewer, succinctly captures and analyzes the interconnectedness of cited authors in this research area. (C) The diagram displays co-occurring authors in liver cancer and single-cell sequencing research, with nodes colored to represent distinct author clusters. Node size indicates the frequency of co-occurrence, and links depict the relationships among co-occurring authors. (D) The map overlays the presentation of heat on the basis of Figure 2C, displaying the frequency of recent publication volume through different shades of color.

The overview of articles published by the main authors in the field was represented by network visualization, where the intensity of colors distinguishes the total number of publications by each author, with darker colors indicating a higher volume of relevant articles (Fig. 2A). Thus, it can be observed from the figure that the overall distribution of publication volume among authors is relatively balanced, with notable contributions from individuals such as Llovet, Jm, and close associations with Zheng, Ch, Zhang, Qm, and Ma, Lc. Co-citation refers to the frequency with which 2 authors are simultaneously cited by third-party authors in the same document. This approach provides intuitive insights into the relevance and similarity of research among authors more intuitively was conducted to analyze the commonality of the authors published articles (Fig. 2B). Based on this, authors in the field of liver cancer single-cell RNA sequencing are primarily classified into 4 clusters (representing 4 research domains): Zhang, Qm, Zheng, Ch, Ma Lc, etc, focusing on liver cancer topics from the perspectives of chemistry, engineering, and materials science (green cluster); authors such as Llovet, Jm, Hoshida, Y, focusing on oncology, hepatology, and cell biology (yellow cluster); Villanueva, A, and Lee, Sung Hak, concentrating on tumor biology and HCC (red cluster); and authors in the blue cluster at the lower left corner, whose research directions lean towards interdisciplinary fields such as gerontology, genetics, and neuroscience, encompassing Sun, Yun-Fun, Ho, Dwh, and others.

The collaboration relationships among authors are shown in different colors, highlighting spontaneously formed clusters of authors engaged in frequent academic exchanges (Fig. 2C). The collaboration relationships among authors, highlighting spontaneously formed clusters that include groups of authors engaged in frequent academic exchanges, shown in different colors. It is worth noting that the distribution of author collaboration clusters generally correlates with their geographical location and country affiliation. However, due to the dominant position of Chinese authors in this field, the majority of the clusters consist of authors from China, leading to a more prominent characteristic of institutional distribution. For instance, in addition to the red cluster, the light blue cluster in the middle, the purple cluster with close connections (including many authors from Nanjing Medical University such as Sun, Guangshun, and Xia, Yongxiang), the green cluster, and the yellow cluster in the lower right corner are primarily composed of Chinese authors. Furthermore, the brown cluster on the left consists of authors from Sun Yat-sen University, such as Hu, Huanjing and Kuang, Ming. The orange cluster in the lower left corner mainly comprises authors from the University of Hong Kong, such as Tsui, Yu-Man, Cheu, Jacinth Wing-Sum, and Cheung, Tan-To. The blue cluster in the upper-right corner mainly consists of authors from the NIH National Cancer Institute in the United States, including Kleiner, David E, Greten, Tim F, and Ma, Lichun. Additionally, there are relatively scattered clusters formed by authors from Japan (e.g., Sakamori, Ryotaro) and Germany (e.g., Tacke, Frank). In addition, the aforementioned collaboration was further analyzed from a temporal perspective (Fig. 2D), with red points representing authors who have engaged in frequent recent academic collaborations, such as Zhou, Jian, Fan, Jia, Wang XueHao, Xia YongXiang, and Zhu Jiye, who have become new research forces.

### 3.4. Instructions and cooperation analysis

**Figure 3.**
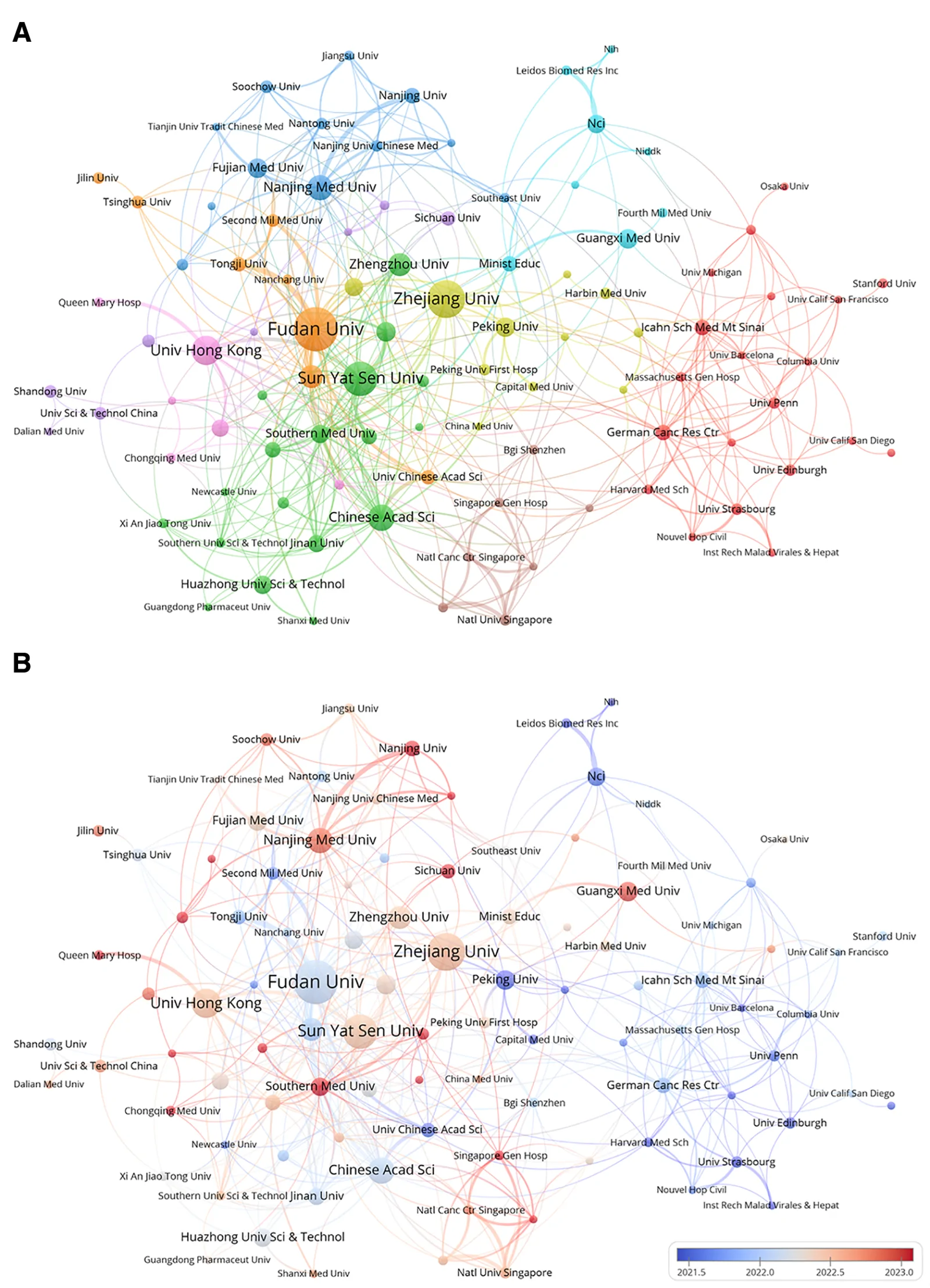
Institutional collaborative network mapping of liver cancer and single-cell sequencing researches. (A) The graph illustrates the co-occurrence of research institutions, with node size representing the frequency of their occurrence together and connections indicating co-occurrence relationships. The size of each node indicates how frequently research institutions collaborate, while the links represent instances of their joint occurrences. (B) The figure illustrates recent contributions of institutions to liver cancer and single-cell sequencing research relative to their overall output. A red bias indicates increased influence, while a blue bias suggests decreased activity in the field. The color scale reflects the ratio of keywords over the past 5 years, highlighting institutions with significant impacts or reduced involvement in this study.

The visualization analysis of collaboration relationships among these institutions highlighted the strong geographical attributes of these collaborations (Fig. 3A). The red cluster on the right primarily consists of institutions from the United States, such as the Icahn School of Medicine at Mount Sinai, Massachusetts General Hospital, and Harvard University. Additionally, it includes some European institutions such as The University of Edinburgh (UK), German Cancer Research Center (Germany), and University of Strasbourg (France), reflecting the complex network of multinational collaboration within this cluster. Other clusters mainly comprise Chinese institutions, with Fudan University holding a central position and maintaining strong collaborative relationships with Tongji University and the University of Chinese Academy of Sciences. Institutions such as Sun Yat-sen University, Zhejiang University, Nanjing Medical University, and The University of Hong Kong also exhibit frequent and extensive academic collaboration with other Chinese institutions. Furthermore, collaboration relationships with temporal distribution characteristics revealed institutions from European countries and the United States, such as the German Cancer Research Center, University of Edinburgh, Icahn School of Medicine at Mount Sinai, and University of Pennsylvania, have established extensive single-cell sequencing and liver cancer research in the early years (Fig. 3B). whereas, the relatively late emergence of academic exchanges between Nanjing Medical University and Nanjing University, as well as the strengthened external academic collaborations of institutions such as Southern Medical University and Sichuan University in recent years (Fig. 3B).

### 3.5. Journal analysis

**Figure 4.**
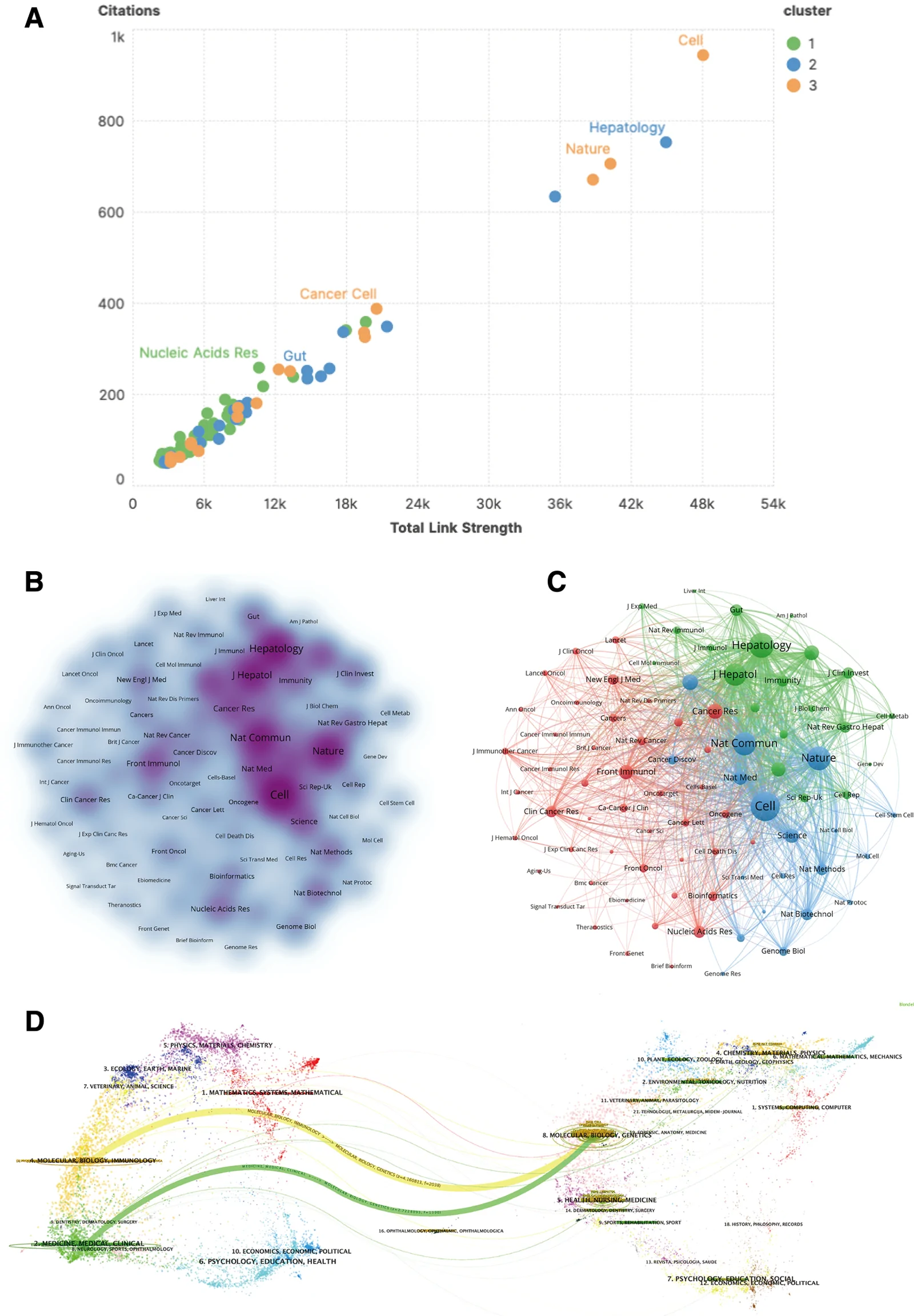
Network visualization of journal publication volume, collaboration, and citation relationships in the field of liver cancer and single-cell sequencing. (A) The scatterplot displays the citation count and total link strength of journals, categorized into 3 clusters based on their collaboration relationships, corresponding to the clustering classification in Figure 4C. (B) The visualization overlays the publication volume of journals, with color intensity indicating the volume of publications. (C) Visualization analysis of the collaboration network of journals in VOSviewer. Journals in different clusters are distinguished by nodes of different colors, with node size representing their frequency of occurrence. (D) The dual-map visualizes journals related to hepatocellular carcinoma single-cell RNA-seq research, where clusters of citing journals are shown on the left side and cited journals on the right side. Colored trajectories between them represent citation relationships.

The citation frequency and total connection strength, which were performed by scatterplot and heatmap, respectively, were used to reveal the research impact of journal. The journal with the highest citation count is *Cell* (944 times), followed by *Hepatology* (753 times) and *Nature* (706 times). Other important journals with citation frequencies exceeding 500 times include *Nature Communications* (671 times) and *Journal of Hepatology* (634 times) (Figure 4A, B). Based on the collaboration relationships among these journals, they are categorized into 3 clusters of different colors, as shown in Figure 4C. Articles in this field are predominantly published in journals focusing on oncology, genetics, and bioinformatics (red cluster, corresponding to cluster 1 in Figure 4A), such as *Frontiers in Immunology* (also the journal with the highest publication volume in this field, with up to 28 articles), *Cancer Research*, *Clinical Cancer Research*, *Nucleic Acids Research*, and *Bioinformatics*. The blue cluster in the lower right corner (corresponding to cluster 3 in Figure 4A) mainly comprises journals in biology and interdisciplinary fields, including well-known top journals in the scientific community such as *Cell*, *Science*, *Nature*, and its subsidiary journal *Nature Communications*.

The co-citation relationships among these journals were also performed and visualized via CiteSpace. Based on the similarity of research content derived from co-citation relationships among journals, these journals are briefly categorized into 4 types (Figure S3A, Supplemental Digital Content 3). The blue cluster on the far left primarily consists of journals in hepatology, such as *Hepatology*, *Hepatology Communications*, and *Nature Reviews Gastroenterology* and *Hepatology*. The red cluster on the right mainly includes journals exploring HCC from the perspectives of oncology and immunology, such as *Frontiers in Immunology*, *Frontiers in Oncology*, and *Cancers*. The green cluster at the bottom includes journals focusing on cell biology and clinical or bioinformatics research, such as *Advanced Science*, *Scientific Reports*, *Cell*, and *Briefings in Bioinformatics*. The yellow cluster in the middle includes journals covering topics in gastroenterology, oncology, cell biology, and immunology, such as *Journal for Immunotherapy of Cancer*, *Gut*, and *Journal of Hematology and Oncology*. Furthermore, time-overlapping analysis found that journals such as *Cancers*, *Apoptosis*, and *Journal of HCC* have shown rapid development in recent years (Figure S3B, Supplemental Digital Content 3). Nevertheless, prestigious journals, like *Nature*, *Cell*, and *journal of Immunotherapy of Cancer,* have published numerous research studies and have a lasting popularity.

According to the annual change in the popularity of journals with higher correlations grouped, we classified the most relevant journals and showed them in a heatmap (Figure S3C, Supplemental Digital Content 3). The timeline in the bottom row indicates the years. Journals such as *Cancer Letters*, *Hepatology Communications*, *Journal of Hepatology*, and *Frontiers in Oncology* were popular before and in 2020. Journals that have shown good development in recent years include *Cancers*, *Journal of HCC*, *Journal of Translational Medicine*, and *Journal of Cancer Research and Clinical Oncology*. Figure 4D shows a dual-map overlay of journals, providing a more intuitive representation of citation relationships among journals and changes in research focus. Citation relationships are indicated by lines connecting citing journals on the left to cited journals on the right. The 2 prominent citation relationships highlighted in the figure indicate that journals focusing on molecular biology, immunology, medicine, and clinical research mainly cite articles from journals in the molecular biology and genetics fields.

### 3.6. Keywords analysis

**Figure 5.**
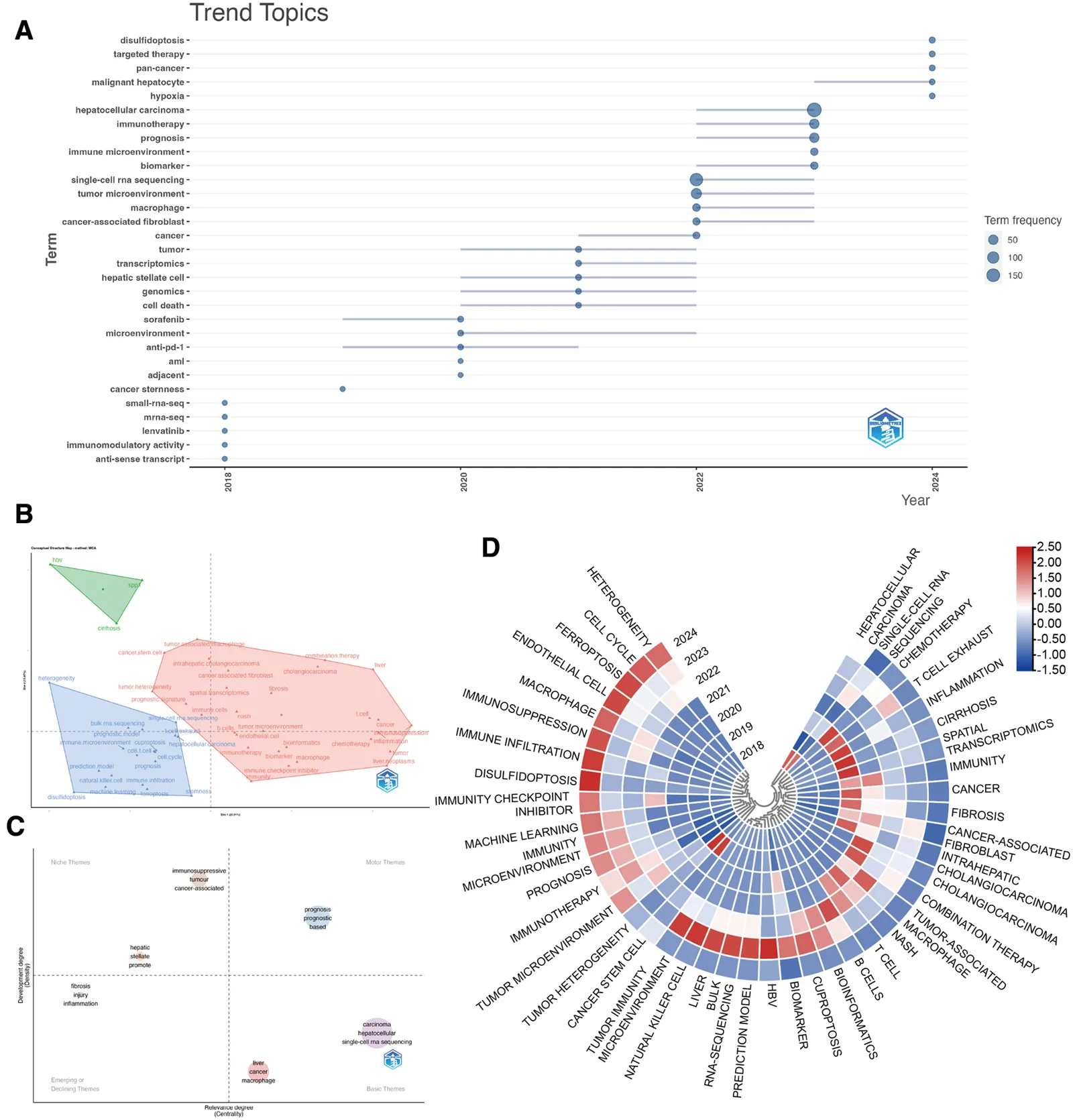
Keywords co-occurrence network mapping and correlation analysis of liver cancer and single-cell sequencing. (A) The visualization analysis of the top thirty keywords by frequency over time depicts the duration of keyword popularity with line length and the frequency of occurrence with dot size, organized chronologically. (B) Using MCA analysis to visualize keyword similarity, the plot identifies 3 main clusters based on keyword distances and color differentiation, indicating higher correlation within each cluster. (C) The x-axis represents relevance, while the y-axis indicates development level. Keywords in the upper-right quadrant show the highest relevance and significant development. (D) The heatmap displays keyword popularity in heat stroke research, categorizing keywords into distinct groups based on their popularity within similar timeframes and differentiated by color.

Keywords, serving as a guide to the direction or a condensed summary of a research article, typically encapsulate the essence of the main topic. Therefore, keyword analysis is an important component of bibliometric analysis, crucial for understanding the current status and predicting future trends in a research field. The trend of keyword occurrence frequency from 2018 to 2024 is depicted in Figure 5A. The length of the lines on the horizontal axis indicates the duration of keyword popularity, while the size of the dots represents the frequency of keyword occurrence. It can be observed that the newly emerged keyword HCC has the highest popularity among keywords from 2022 to 2024 (182 occurrences), followed by single-cell RNA sequencing (134 occurrences), tumor microenvironment (64 occurrences), immunotherapy (49 occurrences), and prognosis (48 occurrences). Subsequently, these keyword charts were categorized and displayed in the form of a cluster graph (Figure S4, Supplemental Digital Content 4). The green cluster on the far left includes terms such as HPV (human papillomavirus), cirrhosis, and spp1 (secreted phosphoprotein 1). The blue cluster, containing the most keywords, primarily consists of terms from the fields of oncology, molecular biology, and immunology, such as immunotherapy, macrophage, and tumor microenvironment. The red cluster on the right comprises terms mainly from bioinformatics, biotechnology, and cell biology, such as single-cell RNA sequencing, natural killer cells, and machine learning.

The co-occurrence relationships and strengths of keywords, aiming to explore potential connections and hot trends among keywords. The core keywords in this field are HCC and single-cell RNA sequencing, with the highest co-occurrence strength (Figure S5A, Supplemental Digital Content 5). Meanwhile, keywords are divided into 8 clusters based on their associations: the green and light blue clusters, centered around HCC, primarily contain immunology and cell biology-related keywords, such as immune infiltration, cell cycle, and B cells; the yellow cluster, centered around single-cell RNA sequencing, also includes some biological analysis-related keywords, such as RNA-seq, WGCNA, and machine learning; the pink and purple clusters mainly consist of keywords from the bioinformatics field, such as prognostic model, data integration, and risk model; the red and orange clusters contain mostly oncology-related terms, such as cancer, liver neoplasms, cancer stem cell, and tumor-associated macrophage; the dark blue cluster encompasses keywords from clinical and cell biology fields, such as combination therapy and cholangiocarcinoma. Combining Figure S5B, Supplemental Digital Content 5, terms such as T cell exhaustion, machine learning, spatial transcriptomics, and angiogenesis emerge as currently popular keywords, which may have significant implications for exploring the mechanisms, interventions, and novel research approaches in HCC.

Subsequently, a heatmap analysis of keywords in the field of 9 HCC single-cell RNA sequencing was constructed, dividing them into 6 clusters based on their relevance and popularity (Figure S5C, Supplemental Digital Content 5). From top to bottom, the first 2 clusters mainly consist of terms related to cancer and single-cell sequencing, such as cancer stem cell and single-cell RNA sequencing. The subsequent clusters contain keywords associated with immunology, including immunity, inflammation, and biomarkers. The next cluster, which contains the most keywords, focuses primarily on cell biology, such as macrophage, ferroptosis, and endothelial cells. The clusters below are closer to clinical terms, such as NASH and cholangiocarcinoma. The bottom cluster includes keywords related to oncology, bioinformatics, and analytical techniques, such as tumor microenvironment, bulk-RNA sequencing, and bioinformatics.

Furthermore, the similarity between keywords was visually depicted through Monotone Comparative Analysis, which represents their distances on the graph and distinguishing them into 3 main clusters using colors to clarify the similarities and differences among the keywords (Fig. 5B). This graph shares similarities with Figure S5C, Supplemental Digital Content 5. The green cluster in the top left corner includes 3 keywords highly related to tumors. The red cluster on the right side encompasses a wide range of clinical and immunological terms. The keywords in the blue cluster in the bottom left corner focus more on bioinformatics and sequencing technology. Following, a concise presentation of the trends in discussion intensity of key terms since 2018 from a temporal perspective was depicted to change of hotspots (Fig. 5C). The horizontal axis represents the frequency or intensity of key term occurrences, while the vertical axis serves as a measure of time, presenting some important keywords in 4 quadrants. Terms like prognosis in the top right corner are popular keywords that have appeared later and have high intensity. Terms such as liver, carcinoma, and macrophage in the bottom right corner are keywords that have sustained research intensity to date. Terms like hepatic and immunosuppressive in the top left corner may represent potential future hotspots. The heatmap in Figure 5D provides a more detailed analysis of the evolution trends of the research hotspots mentioned above. Keywords such as Sequencing, chemotherapy, and tumor heterogeneity were popular research directions before 2020 but have seen a decrease in research intensity in recent years. In contrast, keywords like tumor immunity microenvironment, bulk-RNA sequencing, prediction model, disulfidptosis, and immune infiltration are emerging into focus in recent years.

### 3.7. Cited references analysis

**Figure 6.**
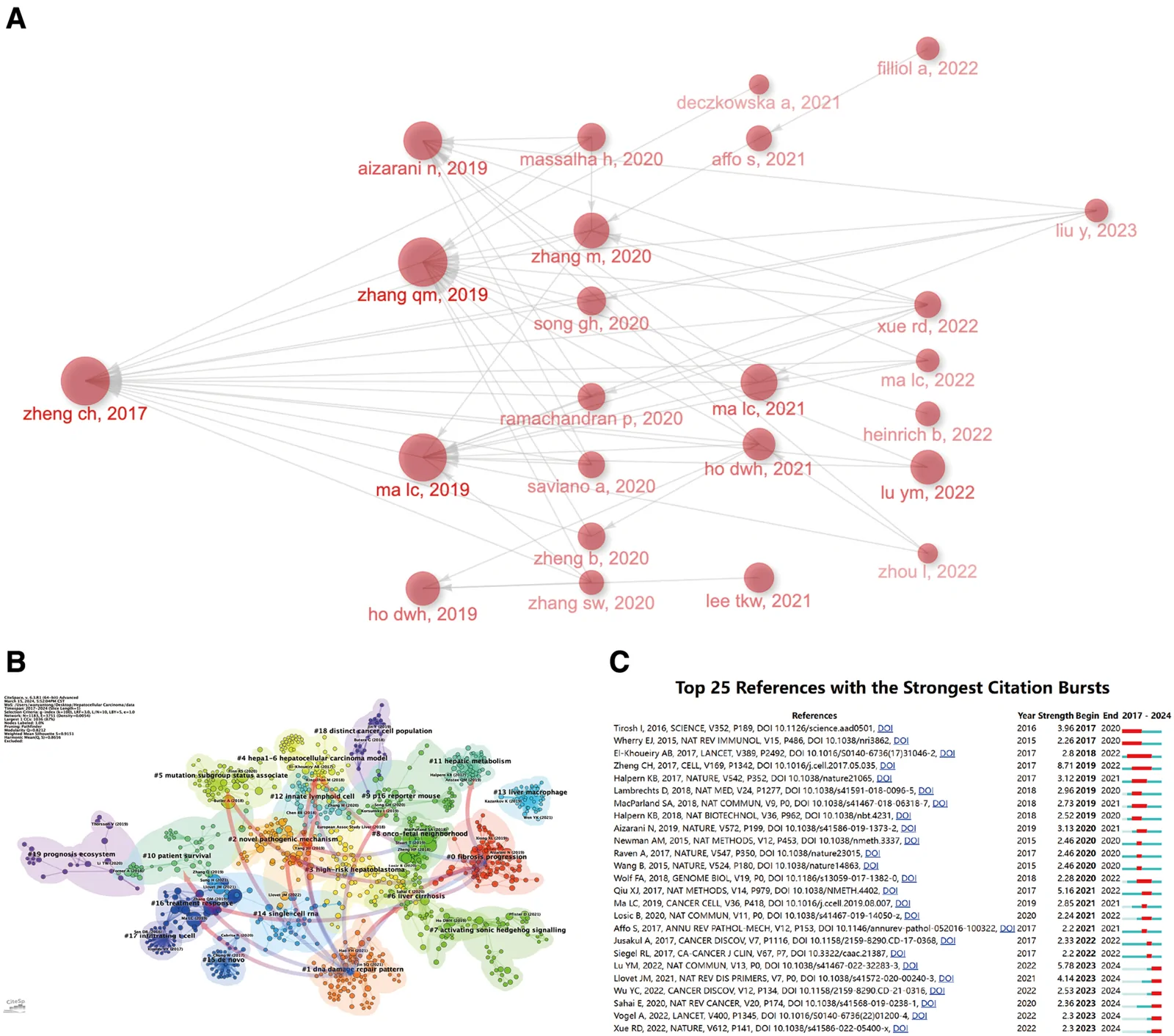
Highly cited references co-citation network mapping and burst of references of liver cancer and single-cell sequencing. (A) The connectivity among the primary 24 citation bursts is demonstrated, depicting the citation interconnections among these articles with arrows. (B) References are grouped based on their likeness, where smaller numbers denote larger clusters. Node size reflects the frequency of co-citations, while the connections between nodes depict co-citation associations. (C) The 25 most cited references.

Citation frequency is an important metric for assessing the quality and impact of research articles, reflecting key factors driving research progress and indicating trends in the field. Among the most highly cited articles in this field is “Landscape of Infiltrating T Cells in Liver Cancer Revealed by Single-Cell Sequencing” published by Zheng, Ch et al in *Cell* in 2017 (1241 citations) (Fig. 6A). This study utilized single-cell RNA sequencing to analyze T cells, particularly tumor-infiltrating lymphocytes, in patients with liver cancer. It aimed to elucidate the functions and mechanisms of infiltrating T cells in the development and progression of liver cancer, providing valuable transcriptomic data resources for future researchers in liver cancer studies.^[9]^ Following closely is “Landscape and Dynamics of Single Immune Cells in HCC,” also published in *Cell* in 2019 by Zhang, Qm et al (732 citations). This study employed 2 single-cell RNA sequencing techniques, SMART-seq2 and high-throughput droplet sequencing (Drop-seq), to analyze CD45 immune cells in multiple tissue organs of patients with liver cancer. It aimed to discover the interactions between immune cells and the tumor microenvironment, their functions in inflammation, and cancer prognosis, thereby exploring new targets for liver cancer immunotherapy or potential biomarkers.^[19]^ Another highly cited article, with over 500 citations, is “A human liver cell atlas reveals heterogeneity and epithelial progenitors” by Aizarani, N et al published in Nature in the same year (573 citations). This study primarily utilized mCEL-Seq2 to perform single-cell RNA sequencing on epithelial progenitor cells in the healthy human liver, providing important theoretical and experimental support for the development of organoids and humanized liver-chimeric mouse models for liver cancer treatment.^[20]^

Figure 6A also illustrates the co-citation network of highly cited articles, emphasizing the groundbreaking contributions of the 2017 paper by Zheng, Ch et al titled “Landscape of Infiltrating T Cells in Liver Cancer Revealed by Single-Cell Sequencing,”^[9]^ which provided a seminal framework for subsequent research in the field. Two years later, “Landscape and Dynamics of Single Immune Cells in HCC” further advanced the application of single-cell RNA sequencing technology in liver cancer research, inspiring more scholars to delve deeper into exploration.^[19]^ Ultimately, these studies were referenced in the 2023 article “Identification of a tumor immune barrier in the HCC microenvironment that determines the efficacy of immunotherapy” by Liu, Y et al.^[21]^ Furthermore, the potential interrelation among the main themes of these studies was demonstrated in Figure 6B, where the high-cited articles are clustered into 20 groups based on their relevance. The most extensively researched topic is fibrosis progression. Early studies focused on keywords such as prognosis, ecosystem and patient survival, which were closely intertwined and evolved into popular topics like novel pathogenic mechanisms, high-risk hepatoblastoma, and DNA damage repair pattern.

Citation burst is an indicator of an active research area, where a reference can sustain a high citation frequency for multiple years. (Fig. 6C). The blue and red lines indicate the cited frequency from 2017 to 2024 and the period of burst-range cited frequency, respectively. We could observe that the article “Landscape of Infiltrating T Cells in Liver Cancer Revealed by Single-Cell Sequencing” published by Zheng, Ch et al in *Cell* in 2017 (8.71 burst citation) was the most cited reference, same as the analysis of co-citation network of highly cited articles (Fig. 6A). The second most cited reference was “A single-cell atlas of the multicellular ecosystem of primary and metastatic HCC” written by Lu YM et al in 2022 (5.78 burst citation). Following closely was “Reversed graph embedding resolves complex single-cell trajectories” written by Qiu XJ et al in 2022 (5.16 burst citation). The high burst of references in recent 2 years included 6 articles, all of which were published in authoritative journals, such as *Nature Reviews Disease Primers*, *Lancet*, and *Nature*.

## 4. Discussion

Emerging single-cell sequencing techniques could reveal gene variation and epigenetic modification at the molecular level of the genome, transcriptome, and epigenetics at the single-cell level, and could also be used to identify new cell types and surface markers.^[22]^ Single-cell sequencing breaks through the bottleneck of previous experimental techniques and can reflect the heterogeneity between cells, which is conducive to revealing the mechanism of disease.^[23]^ The liver has complex cell stages with different cell composition and differentiation degrees, and it is easy to produce hepatitis under the influence of pathogen infection, drugs, metabolic disorders, and abnormal immune regulation.^[24]^ The liver function may gradually deteriorate in the state of continuous inflammation, leading to cirrhosis or liver cancer.^[24]^ However, the thorough understanding and precise treatment of liver cancer are very limited. The emergence of single-cell sequencing technology is of great significance to reveal the internal pathogenesis of liver cancer, which enables research to understand the etiology of liver cancer and the physiological and pathological significance related to this disease from the molecular level, and provides a powerful means for the study of clinical molecular typing, specific targets and precise treatment.^[25]^ In this study, we systematically analyzed the published articles on single-cell sequencing and liver cancer through bibliometric analysis to find the research hotspots, trends, research cooperation relationship and future development in this field.

Looking back at its history, the first example of single-cell RNA sequencing was invented by Tang et al in 2009.^[6]^ Whereas single-cell sequencing research in liver cancer began in 2017, Zheng, Ch et al published the landmark article in *Cell* “Landscape of Infiltrating T Cells in Liver Cancer Revealed by Single-Cell Sequencing.”^[9]^ Since then, the vigorous development of research in this field has promoted the basic research and clinical application of single-cell sequencing in liver cancer. The trend of article publication and citation can be seen that the published articles in the past 3 years show a steep rise. The polynomial fitting of the cumulative number of publications over the years also indicates vigorous research activity in this field in the future. We speculate that the continuous publication of articles in this field provides a rich resource reference for the research of single-cell sequencing on liver cancer, and improving our understanding of tumor biological characteristics.

Collaborative research has become increasingly valued across scientific disciplines, and this field is no exception. Our bibliometric analysis reveals that China and its domestic institutions dominate the field of single-cell sequencing in liver cancer research in terms of publication output and collaborative networks. This dominance is unlikely to be coincidental. Instead, it likely reflects a convergence of several factors. First, China bears a disproportionate burden of liver cancer, accounting for nearly half of global cases and deaths, which creates an urgent clinical need for innovative diagnostic and therapeutic strategies.^[26]^ Second, substantial and sustained investment from Chinese funding agencies, such as the National Natural Science Foundation of China, has prioritized precision medicine and high-throughput sequencing technologies, enabling the rapid establishment of single-cell core facilities and training of computational biologists.^[27]^ Third, the large patient population facilitates the collection of fresh tissue samples: a critical requirement for high-quality single-cell sequencing, allowing Chinese research groups to generate large-scale, clinically annotated single-cell atlases.^[28]^ This leadership position has important implications for global collaboration: while China has established extensive partnerships with the United States and European countries, the field would benefit from more balanced, bidirectional exchanges where data standards, analytical pipelines, and biological insights are shared equitably to accelerate worldwide progress against liver cancer.

Unlike previous reviews that have qualitatively summarized technical applications of single-cell sequencing in liver cancer,^[12]^ the present study provides a systematic bibliometric analysis that quantitatively maps the evolution, hotspots, and future trends of this field. Notably, while prior reviews have highlighted immunotherapy and the tumor microenvironment as important areas,^[29]^ our analysis uniquely employs citation burst detection to predict that “machine learning” and “bulk RNA-seq integration” will become dominant research forces over the next 3 years: a quantitative foresight absent from previous reviews. Furthermore, unlike existing bibliometric studies that have often limited their scope to publication counts and collaboration networks,^[13]^ our study integrates co-citation analysis with temporal keyword evolution, revealing a paradigm shift from descriptive phenotyping to predictive modeling. This data-driven roadmap distinguishes our work and provides an evidence-based direction for future research.

The main task of this study is to find the hot spots and trends of single-cell sequencing and liver cancer, and the analysis of keywords could provide us with rich information. In general, the frequency of keyword occurrence was diverse in different time periods. And the co-occurrence relationships of keywords showed that single-cell sequencing for liver cancer presented multiple fields. In addition to recent high-frequency keywords of HCC and single-cell RNA sequencing, keywords such as immunotherapy, immune infiltration and tumor microenvironment were also hotspots in research, indicating that the field of immunity was the research trend. Besides, the temporal perspective of keywords revealed that immunosuppression may potential future hotspots. Interestingly, as the reference with the highest citation intensity, “Landscape of Infiltrating T Cells in Liver Cancer Revealed by Single-Cell Sequencing” published by Zheng, Ch et al in *Cell* devoted to describing the immune landscape of Liver cancer, was even published in 2017.^[9]^ Single-cell sequencing of liver cancer remains a hot topic in immunology, particularly the immunotherapy, immunotyping and immune targets of liver cancer are ongoing investigations.^[25]^ For example, the article published in *Nature* in 2022 written by Ning Zhang et al “Liver TIME subtypes and neutrophil heterogeneity” systematically revealed the immune microenvironment subtypes of liver cancer, the functional heterogeneity of tumor-associated neutrophils, and tumor-associated neutrophils as a new immunotherapy strategy for liver cancer.^[30]^ Zhang et al combined single-cell sequencing with spatial transcriptomics to determine the spatial structure of the tumor immune barrier in TIME and explore whether tumor immune barrier determined the response of patients with HCC to immunotherapy, which was published in *the Journal of Hepatology,* “Identification of a tumor immune barrier in the HCC microenvironment that determines the efficacy of immunotherapy” in 2023.^[21]^ Therefore, the field of immunity is the hotspot for single-cell sequencing of liver cancer in the future.

The heatmap of the evolution trends of the research hotspots of keywords found that bulk-RNA sequencing and prediction models were emerging into focus in recent years. These keywords were related to single-cell sequencing combined with multi-omics analysis, machine learning, and multi-dimensional bioinformatics, which were a strong research force in recent years.^[31]^ What attracted our attention was that bioinformatics analysis based on big data and machine learning was a research methods that have emerged recently, and the research on liver cancer was no exception. Big data-machine learning was used to build a model to optimize imaging examination, predict the risk of liver cancer occurrence and postoperative recurrence and survival, and analyze the protein expression of liver cancer.^[32]^ These research methods benefit from the vigorous development of big data storage, artificial intelligence development, and data network interconnection in recent years, providing a new research method for single-cell sequencing in liver cancer research. Based on the heatmap of the evolution trends of the research hotspots, we speculate that future research directions should focus on integrating single-cell RNA sequencing with bulk-RNA-seq, multi-omics analysis, machine learning, and multi-dimensional bioinformatics to elucidate the pathogenesis of liver cancer and identify clinical therapeutic targets. By combining the high-resolution cellular insights from single-cell data with the cohort-level statistical power of bulk-RNA-seq, and leveraging machine learning algorithms to integrate genomic, epigenomic, proteomic, and transcriptomic layers, researchers can deconvolve tumor heterogeneity, predict patient prognosis and treatment responses, and discover novel biomarkers or drug targets.^[33,34]^ This integrative approach holds great promise for translating single-cell discoveries into clinically actionable strategies, ultimately advancing precision medicine for liver cancer.^[33]^

For the analysis of highly cited references, the article “Landscape of Infiltrating T Cells in Liver Cancer Revealed by Single-Cell Sequencing” published in *Cell* in 2017 by Zheng, Ch et al^[9]^ has been cited the most frequently, providing a leading idea for subsequent research in this field and inspiring more researchers to conduct in-depth investigation. Therefore, this article could be summarized as the trailblazer of single-cell sequencing in liver cancer research. There is no denying that this article has also become the highest citation burst reference. The co-citation network of highly cited articles showed that a considerable part of the highest citation references was published by Chinese authors, and played a role in linking the previous and the next research in this field, which indicated the key role of Chinese scholars in single-cell sequencing on liver cancer research, consistent with the meaning of analysis of cooperative relations of country and author.

Despite its advantages, single-cell sequencing in liver cancer research has several limitations. The high cost and requirement for fresh tissue samples limit its clinical scalability.^[35]^ Tissue dissociation may introduce cellular stress and selective loss of certain cell types, leading to representation biases.^[36]^ Additionally, single-cell RNA-seq has lower transcriptome coverage compared to bulk-RNA-seq, resulting in dropout events and reduced sensitivity for low-abundance transcripts.^[37]^ Computational challenges, including high dimensionality and batch effects, further complicate data analysis.^[38]^ Finally, functional validation and translation of single-cell discoveries into clinical applications remain significant hurdles.^[39]^

## 5. Conclusion

The Landscape mapping of single-cell sequencing in liver cancer, based on systematic bibliometric analysis, indicates that this research field will continue to flourish, with more high-quality articles expected to be published. As a new force in this field of research, China has established extensive cooperative relations with other countries, and Chinese scholars will continue to explore research in this field in-depth. Single-cell sequencing in liver cancer involves different research fields and methods, and its research hotspots and trends change with different period, and Big data, bioinformatics analysis, single-cell multi-omics analysis, immunotherapy, and machine learning are recently emerging studies hotspots. In general, it is hoped that this analysis will provide an accurate direction for research in this field and promote the basic research and clinical application of liver cancer.

## Author contributions

**Conceptualization:** Wenbiao Chen.

**Data curation:** Feng Xiong.

**Formal analysis:** Feng Xiong.

**Investigation:** Haitao Li.

**Methodology:** Haitao Li.










